# Length polymorphism and head shape association among genes with polyglutamine repeats in the stalk-eyed fly, *Teleopsis dalmanni*

**DOI:** 10.1186/1471-2148-10-227

**Published:** 2010-07-27

**Authors:** Leanna M Birge, Marie L Pitts, Baker H Richard, Gerald S Wilkinson

**Affiliations:** 1 Department of Biology, University of Maryland, College Park, MD 20742 USA; 2 University College London, Research Department of Genetics, Evolution and Environment, Wolfson House, 4 Stephenson Way, London, NW1 2HE, UK; 3 Department of Biology, The College of William and Mary, Williamsburg, VA 23187 USA; 4 Sackler Institute for Comparative Genomics, American Museum of Natural History, New York, NY, 10024 USA

## Abstract

**Background:**

Polymorphisms of single amino acid repeats (SARPs) are a potential source of genetic variation for rapidly evolving morphological traits. Here, we characterize variation in and test for an association between SARPs and head shape, a trait under strong sexual selection, in the stalk-eyed fly, *Teleopsis dalmanni*. Using an annotated expressed sequence tag database developed from eye-antennal imaginal disc tissues in *T. dalmanni *we identified 98 genes containing nine or more consecutive copies of a single amino acid. We then quantify variation in length and allelic diversity for 32 codon and 15 noncodon repeat regions in a large outbred population. We also assessed the frequency with which amino acid repeats are either gained or lost by identifying sequence similarities between *T. dalmanni *SARP loci and their orthologs in *Drosophila melanogaster*. Finally, to identify SARP containing genes that may influence head development we conducted a two-generation association study after assortatively mating for extreme relative eyespan.

**Results:**

We found that glutamine repeats occur more often than expected by amino acid abundance among 3,400 head development genes in *T. dalmanni *and *D. melanogaster*. Furthermore, glutamine repeats occur disproportionately in transcription factors. Loci with glutamine repeats exhibit heterozygosities and allelic diversities that do not differ from noncoding dinucleotide microsatellites, including greater variation among X-linked than autosomal regions. In the majority of cases, repeat tracts did not overlap between *T. dalmanni *and *D. melanogaster *indicating that large glutamine repeats are gained or lost frequently during Dipteran evolution. Analysis of covariance reveals a significant effect of parental genotype on mean progeny eyespan, with body length as a covariate, at six SARP loci [CG33692, *ptip*, *band4.1 inhibitor LRP interactor*, *corto*, 3531953:1, and *ecdysone-induced protein 75B *(*Eip75B*)]. Mixed model analysis of covariance using the eyespan of siblings segregating for repeat length variation confirms that significant genotype-phenotype associations exist for at least one sex at five of these loci and for one gene, CG33692, longer repeats were associated with longer relative eyespan in both sexes.

**Conclusion:**

Among genes expressed during head development in stalk-eyed flies, long codon repeats typically contain glutamine, occur in transcription factors and exhibit high levels of heterozygosity. Furthermore, the presence of significant associations within families between repeat length and head shape indicates that six genes, or genes linked to them, contribute genetic variation to the development of this extremely sexually dimorphic trait.

## Background

Repetitive, low complexity DNA sequences are ubiquitous in nature [1]. While these sequences are commonly utilized as markers for genetic mapping studies, few of them have been implicated as causal elements of phenotypic change. One class of repetitive sequences, known as single amino acid or codon repeats, is an exception to this pattern and has long been known to be associated with diseases of the nervous system [2,3]. Indeed, variation in the length of single amino acid tracts, often referred to as single amino acid repeat polymorphisms (SARPs), have been implicated in a variety of neuropathologies [2-5], such as Fragile-X [6], Kennedy's disease [7]. Huntington's chorea [8,9] and others [2,10,11]. In some cases, codon repeat length is positively associated with disease severity [2,12,13]. For example, a sequence of 36 or more glutamine repeats in the Huntingtin gene results in a protein product that increases neural decay [14] and causes Huntingtin's chorea [14,15]. Larger glutamine repeat tracts are associated with earlier onset and accelerated progression of the disease [16].

SARPs have also been proposed as a source of genetic variation for rapidly evolving morphological traits [17,18]. This proposition is based on the observation that trinucleotide repeats are common in eukaryotic DNA [19-21] and undergo mutation as a consequence of replication slippage more frequently than amino acid substitutions [11,22] but see [23]. Rather than being purged from the genome, long repeats are frequently conserved across vertebrates [19,24-27] and those containing glutamine or alanine tend to occur in transcription factors [28,29]. Contraction or expansion of a codon repeat in a transcription factor has the potential to modulate gene regulation in a quantitative, rather than qualitative, manner [10,30] and, therefore, result in a mutation of small effect [22]. Thus, SARPs appear to have the potential both to generate genetic variation and to enable adaptive change in morphology.

In this paper we use stalk-eyed flies as a model system [sensu [31]] to determine if SARPs could contribute to the rapid and recurrent evolution of extreme sexual dimorphism for eyestalk length in these flies [32,33]. We designed the study to address four questions: 1) Are any SARPs unequally represented among amino acids in genes expressed during eyestalk development? 2) Are any SARPs preferentially located in transcription factors? 3) Are SARPs gained or lost frequently across species? 4) Do SARPs occur in genes that influence eyestalk length? To increase the likelihood of finding genes with adaptive phenotypic effects, we focus the study on repeats with nine or more consecutive codons because long repeats typically disrupt function and should, therefore, only persist if they provide some selective advantage [17-19,24-27]. Furthermore, because replication slippage typically increases with repeat length [34], long repeats are also likely to exhibit genetic variation.

To identify genes with long repeats we use an annotated expressed sequence tag (EST) database [35] containing over 4,000 unique open reading frames derived from *Teleopsis dalmanni *[recently synonymized with *Cyrtodiopsis*, [36]] brain and eye-antennal imaginal disc tissue dissected from third-instar larvae or 1-7 day-old pupae. During this period of time these tissues develop into the adult head, eyes and brain [37]. To determine if codon repeat frequency is independent of amino acid frequency we use homologous gene regions of *T. dalmanni *and *Drosophila melanogaster*. To assess bias in gene function we compare genes with codon repeats to all genes in the *T. dalmanni *annotated library [35]. Using an outbred population of flies we quantify allelic diversity and heterozygosity for a sample of genes containing polyglutamine repeats and compare them to the same metrics scored on the same flies for a sample of noncoding dinucleotide microsatellites [38]. We made this comparison to determine if length variation in codon repeats is comparable to noncodon repeats, as would be expected if they mutated by a common mechanism and experienced similar constraints. We assess the frequency with which amino acid repeats may be gained or lost by finding all long repeats in homologous gene regions of both *T. dalmanni *and *D. melanogaster *and then determining if a repeat is present in the ortholog. Finally, we conduct a two-generation association study to determine if parental genotype at 32 SARP loci predicts offspring phenotype after assortatively mating by relative eyespan. To confirm significant associations at candidate loci we test for differences in eyespan among genotypes in siblings that differ in repeat length at each candidate locus.

## Results

### Distribution of codon repeat loci

**Figure 1:**
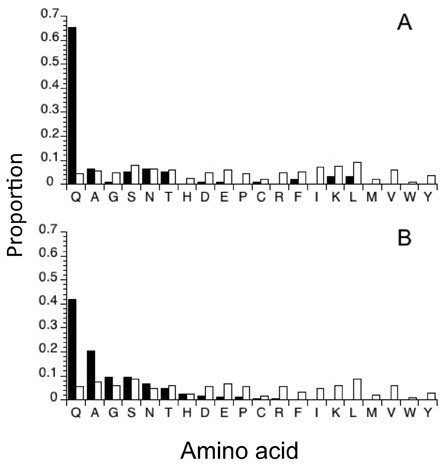
**Distribution of single amino-acid repeats containing more than 8 consecutive residues (filled bars) plotted with the relative abundance of each amino acid (open bars) for two fly species**. Panel A: Proportion of 98 unique open reading frames containing SARs identified in the *Teleopsis dalmanni *EST database. Panel B: Proportion of 343 genes containing SARs in regions of *Drosophila melanogaster *genes homologous to the *T. dalmanni *EST database.

To determine if any repeats containing nine or more codons occur out of proportion to codon abundance, we compared repeat frequencies to corresponding amino acid frequencies for genes in the *T. dalmanni *EST library and their homologs in *D. melanogaster *(Fig. 1). We found that repeat abundance varies independently of amino acid abundance in each species (*T. dalmanni: *χ^2 ^= 895.9, df = 19, P < 0.0001; *D. melanogaster: *χ^2 ^= 1064.6, df = 19, P < 0.0001). For both species, glutamine (Q) occurred in repeats much more often than any other amino acid (Fig. 1). In *T. dalmanni*, no other amino acid was over represented in repeats, but three amino acids - isoleucine (I), valine (V), and arginine (R) - formed repeats less often than expected. In *D. melanogaster *two other amino acids (A and G) were over represented, six amino acids (S, N, T, H, C and W) occurred in proportion to their abundance and the remaining 11 amino acids were under represented in repeats.

### Function of codon repeat loci

We used GeneMerge [39] to determine if the molecular functions of genes with repeats represent a nonrandom sample of the EST library. This analysis revealed that the 98 unique genes with codon repeats were more likely to exhibit DNA binding (P = 0.0021) or transcription regulator activity (P = 0.0092) after Bonferroni correction than expected. When this GeneMerge analysis was repeated using only the 64 genes that carry glutamine repeats, similar results were obtained: RNA polymerase II transcription factor activity (P = 0.0069), transcription regulator activity (P = 0.012), and DNA binding (P = 0.015) were the only molecular functions that were over-represented in the sample.

### Length variation in codon repeats

To quantify standing genetic variation in codon length we genotyped a large sample of flies reared from an outbred population of *T. dalmanni *originally collected in 1999 in peninsular Malaysia [40,41]. Length polymorphism was detected at 25 of 32 glutamine repeat loci (Table 1). Observed heterozygosity, (average ± SE) 0.48 ± 0.03, was similar to allelic diversity, 0.53 ± 0.03, across loci. Nevertheless, after Bonferroni adjustment, goodness-of-fit tests revealed that genotype frequencies at seven loci deviated significantly from expectation (Table 1). At five loci (CG12104, CG31224, *Eip75B*, *M-spondin*, and *toutatis*) there was a deficiency of heterozygotes while at two loci (*corto *and CG10082) there were more heterozygotes than expected.

**Table 1:** Heterozygosity and allelic diversity of glutamine repeat loci in *T. dalmanni*

Locus (chromosome*)	**H**_**o**_	**H**_**e**_	χ^2^	P	**Allele No**.	N
Band4.1 inhibitor LRP (2)	0.59	0.59	0.00	ns	4	163
Bifocal (1)	0.73	0.65	2.75	ns	4	91
Bunched (X)	0.70	0.66	0.62	ns	3	92
Cap-n-collar (2)	0.48	0.50	0.08	ns	2	91
CG10082 (2)	0.67	0.53	7.04	0.0080	4	90
CG10321 (2)	0.63	0.60	0.38	ns	3	90
CG10435 (2)	0.54	0.41	0.08	ns	2	91
CG12104 (1)	0.31	0.45	7.31	0.0069	2	91
CG17265	-	-	-	ns	1	94
CG31064 (2)	0.51	0.50	0.07	ns	5	165
CG31224 (2)	0.30	0.65	38.03	< 0.0001	4	71
CG33692 (1)	0.61	0.60	0.16	ns	4	166
CG34347 (2)	0.60	0.60	0.00	ns	6	91
CG42389 (X)	0.56	0.61	1.87	ns	4	165
CG4409 (2)	0.35	0.41	1.13	ns	2	94
CG8668 (X)	0.68	0.70	0.28	ns	5	159
Corto (2)	0.74	0.67	4.13	0.042	5	155
Cryptocephal (X)	0.57	0.59	0.22	ns	6	167
Cyclin-dependent kinase 8	-	-	-	ns	1	94
Dachshund	-	-	-	ns	1	94
Dorsal switch protein 1	-	-	-	ns	1	94
E5 (2)	0.47	0.42	0.65	ns	2	86
Ecdysone-induced protein 75B (1)	0.12	0.47	43.53	< 0.0001	2	92
M-spondin (2)	0.15	0.32	12.38	0.0054	4	89
Mastermind (2)	0.26	0.25	0.00	ns	3	90
Mediator complex subunit 26	-	-	-	ns	1	94
Ptip (1)	0.50	0.53	0.40	ns	5	90
Sine oculis-binding protein	-	-	-	ns	1	94
SRPK (2)	0.60	0.60	0.00	ns	3	75
Tenascin major (1)	0.28	0.30	0.15	ns	2	92
Toutatis (2)	0.46	0.61	8.50	0.0063	7	167
3531953:1 (X)	0.64	0.59	1.55	ns	5	163

*chromosome identity corresponds to Johns et al. (2005)

SARP loci were assigned to chromosome based on evidence of linkage to 15 noncoding microsatellite markers, which were genotyped for the same sample of flies as the SARP loci, and had previously been located on chromosomes by linkage mapping [42]. Among these flies the noncoding microsatellites had 2-6 alleles and observed heterozygosities ranging from 0.135 to 0.744. Chi-squared contingency tests on genotype counts of noncoding microsatellite markers and SARP loci revealed that eight SARP loci were associated with the first chromosome while 16 SARP loci were associated with the second chromosome and five were associated with the X chromosome (Table 1). Thus, 17% of SARP loci were found on the X chromosome, consistent with the relative size of the X estimated by other methods [43].

In a previous analysis of genetic variation among anonymous noncoding microsatellites [38], X-linked markers exhibited greater genetic variation than autosomal markers. Consequently, we compared genetic variation at glutamine repeat loci to noncoding microsatellites by type of chromosome. A two-way ANOVA on observed heterozygosity revealed that type of chromosome (F_1,43 _= 5.60, P = 0.023), but not type of repeat (F_1,43 _= 0.09, P = 0.76), was significant. Loci on the X chromosome exhibited greater heterozygosity (0.61 ± 0.05) than autosomal loci (0.46 ± 0.03). Similar results were obtained for expected heterozygosity, i.e. type of chromosome (F_1,43 _= 5.61, P = 0.022), but not type of repeat (F_1,43 _= 0.0003, P = 0.99), was significant. In contrast, the average number of alleles per locus did not depend on chromosome (F_1,43 _= 0.15, P = 0.70) or type of repeat (F_1,51 _= 0.003, P = 0.96)

We located two or more EST sequences for 12 genes that contained nine or more glutamine residues. After translating the nucleotide sequences we found variation in the length of the glutamine tract for ten of these genes. A comparison of those sequence variants with the length variants identified by PCR revealed that the length variants found among the flies that were genotyped corresponded to an ORF length as predicted by the EST sequences in all but one case (Table 2). For *dorsal switch protein 1 *there were two length variants among the EST sequences but only a single length variant was identified by PCR.

**Table 2:** Amino acid sequence variants in the *T. dalmanni *EST database with length variants obtained by PCR.

Gene	EST sequences	Repeat length	Repeat sequence	PCR product length (bp)
CG12104	1	14	QQQQQQQQQQQQQQ	192
	4	13	QQQQ-QQQQQQQQQ	189
CG32133	2	14	QQQQQQQQQQSQQQ	214
	1	10	----QQQQQQSQQQ	202
CG4409	3	19	QQQEQEQQQQQQQQQQQQQ	214
	6	16	QQQEQEQQQQ---QQQQQQ	205
Corto	1	19	QQQQQQQQQQQYQQQQQQQ	496
	1	18	QQQQQQQ-QQQYQQQQQQQ	493
Cryptocephal	2	27	QQQQQQQQQQQQQQQQQQQQQQQQQQQ	227
	2	25	QQQQQQQQQQQQQQQQQ--QQEQQQQQ	221
	1	24	QQQQQQQQQQQQQQQQ---QQEQQQQQ	218
	1	23	QQQQQQQQQQQQQQQ----QQEQQQQQ	215
	4	20	QQQQQQQQQQQQ-------QQEQQQQQ	206
	4	16	QQQQQQQQ-----------QQQQQQQQ	194
Dorsal switch protein 1	1	50	QQQQQQQQQQQQQQQQQQQQQQQQQQQQQQQQQQQQHQQQQQQIQQQQQQ	181
	1	48	QQQQQQQQQQ--QQQQQQQQQQQQQQQQQQQQQQQQHQQQQQQIQQQQQQ	-
Mastermind	2	26	QQQQSQAQQQQQQQQQQQQQQKQQQQ	523
	1	25	QQQQFQA-QQQQQQQQQQQQQKQQQQ	520
SRPK	1	30	QQQRQQQQQQQQQFQQQQQYQQQQQYQQQQ	172
	1	26	QQQRQQQQQQQ----QQQQFQQQQQYQQQQ	160
Tenascin major	2	15	QQQQQQQQQQQQQQQ	206
	1	13	QQQQQQQQQQ-QQQ	200

### Evolution of glutamine repeats

A total of 60 genes had a polyglutamine repeat longer than eight residues in either *T. dalmanni *or *D. melanogaster *(Table 3). The two species had similar numbers of genes with repeats (48 in *T. dalmanni *vs. 45 in *D. melanogaster*), although there were substantial differences between the species in the location of the repeats. Of the 84 total repeats found, only nine occurred in homologous regions in both species (in the genes *dachshund*, *dorsal switch protein 1, CG17271, corto, cyclin-dependent kinase 8, mastermind, pumilio *and *scribbler*). In contrast, 39 repeats (46%) have a homologous counterpart that contains 2 or fewer glutamines in the other species.

**Table 3:** Glutamine content for aligned gene regions in *D. melanogaster *and *T. dalmanni*

Gene name	Glutamine #		Gene name	Glutamine #	
	*Dm*	*Td*		*Dm*	*Td*
Band4.1 inhibitor LRP interactor	7	9	dikar	16	2
big brain	13	2	domino	4	16
bunched	4	18	Dorsal switch protein 1	22	36
cap-n-collar	9	12	E2F transcription factor	9	5
CG10082	1	10*	E5	3	10
CG10082	2	19**	E5	7	10
CG10321	3	16	E5	0	9
CG10321	1	9	Ecdysone-induced protein 75B	9	2
CG12104	1	14	grainy head	9	0
CG12488	9	3	grainy head	9	1
CG14023	16	1	GUK-holder	1	9
CG14023	12	1	GUK-holder	1	12
CG14213	12	1	hairy	6	10
CG14440	9	2	headcase	10	0
CG14441	16	12*	headcase	20**	5
CG14441	10	2	jim	17	0
CG14650	17	14	La related protein	4	9
CG17265	1	14	mastermind	14	0
CG17271	10	10	mastermind	17	7
CG17446	21	9*	mastermind	12*	21
CG17446	12	4	mastermind	5	10
CG2083	8	9	mastermind	12*	13
CG31064	7	11	mastermind	14	14
CG31738	0	15	Mediator complex subunit 26	2	14
CG32772	9	3	milton	0	9
CG34114	9	1	M-spondin	0	11
CG34114	8	10	M-spondin	6	9
CG34347	0	11	pipsqueak	12	9*
CG4068	2	9	Protein associated with topo II related - 1	5	9
CG4702	1	9	ptip	35	1
CG5053	12	5	ptip	7	10
CG6619	23	12*	pumilio	13	11
CG8668	2	9	pumilio	12	15
Cirl	10	6	Regena	9	2
corto	17	0	reversed polarity	9	0
corto	8	10	reversed polarity	9	0
corto	11	10	scribbler	21	22
cryptocephal	0	27	scribbler	10	5
C-terminal Src kinase	9	4	Sine oculis-binding protein	0	12
Cyclin-dependent kinase 8	27	27	SRPK	2	9
dachshund	11	11	Tenascin major	1	13
dachshund	15	4	wallenda	2	9

*Region does not contain a run of 9 consecutive glutamines.

**Region contains two polyglutamine repeat regions separated by a single non-glutamine amino acid.

### Association of glutamine repeat length and eyespan

To identify SARP loci with potential effects on relative eyespan we tested for an association between genotype and phenotype after one generation of artificial selection by measuring relative eyespan for 587 flies, selecting extreme males and females, assortatively mating 92 pairs, and collecting their progeny. We then measured a sample of male and female progeny from each of 51 families and tested if parental genotype predicted offspring breeding value for either sex. ANCOVA on average eyespan, with body length as a covariate, for either 10 male or 10 female progeny by parental genotype at each locus revealed five autosomal loci [*band4.1 inhibitor LRP interactor*, *ptip*, CG33692, *corto*, and *ecdysone-induced protein 75B *(*Eip75B*)] in which P < 0.01 for at least one sex (Table 4). In addition, one of five X-linked loci [3531953:1] exhibited a nearly significant effect (P = 0.011) of male parental genotype on female phenotype (Table 5).

**Table 4:** ANOVA on progeny eyespan by parent genotype for autosomal polyglutamine loci

	Female eyespan		Male eyespan		
Locus	F	P	F	P	N
Band4.1 inhibitor LRP interactor	6.01	0.0002	4.72	0.0016	98
Cap-n-collar	0.55	0.58	0.57	0.57	89
CG10082	1.57	0.18	1.77	0.13	88
CG10321	0.8	0.49	1.82	0.13	88
CG10435	0.04	0.85	0.04	0.84	89
CG12104	3.19	0.046	2.09	0.13	89
CG31064	0.35	0.93	0.69	0.68	98
CG31224	1.11	0.37	1.76	0.10	73
CG33692	2.98	0.011	3.16	0.0074	98
CG34347	0.66	0.78	1.01	0.45	89
CG4409	1.43	0.24	1.38	0.26	92
Corto	2.25	0.022	2.59	0.0087	96
E5	0.85	0.43	0.90	0.41	84
Ecdysone-induced protein 75B	2.71	0.07	6.13	0.0032	91
M-spondin	0.37	0.83	0.75	0.56	87
Mastermind	1.52	0.21	2.19	0.08	88
Ptip	1.25	0.28	2.84	0.0079	88
SRPK	1.95	0.06	2.54	0.015	99
Tenascin major	0.56	0.57	1.91	0.15	90
Toutatis	1.07	0.40	1.23	0.28	89

**Table 5:** ANOVA on progeny eyespan by parent genotype for X-linked polyglutamine loci

		Female eyespan		Male eyespan		
Locus	Parent	F	P	F	P	N
Bunched	Male	0.22	0.80	0.42	0.66	45
	Female	0.71	0.62	0.88	0.50	45
						
CG8668	Male	0.65	0.69	0.60	0.73	48
	Female	0.64	0.77	1.18	0.34	46
						
CG42389	Male	2.04	0.12	1.87	0.15	49
	Female	0.81	0.55	1.83	0.13	50
						
Cryptocephal	Male	0.82	0.54	1.28	0.29	50
	Female	0.33	0.92	0.71	0.65	49
						
3531953:1	Male	4.18	0.011	3.03	0.039	48
	Female	0.99	0.46	0.97	0.47	49

**Figure 2:**
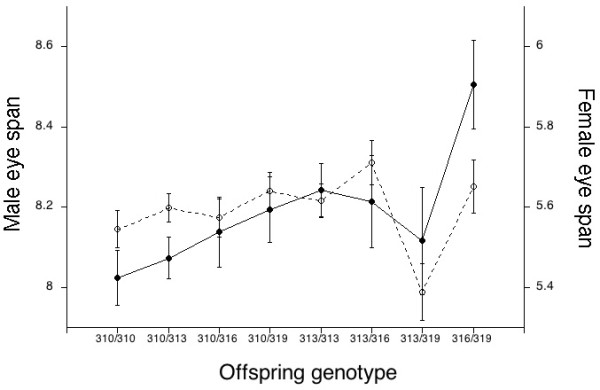
**Least square adjusted mean eyespan for male (solid) and female (dashed) plotted against genotype for progeny from six families that segregated for allelic variants at CG33692**.

To corroborate these putative parental genotype-offspring phenotype associations, we inspected parental genotypes to identify at least five families for each locus in which informative alleles should be segregating among siblings. At one locus, *ptip*, only one such family was found, so that locus was not tested further. For the other five loci, at least 30 progeny of each sex were measured, extracted and genotyped from a total of 19 informative families. A mixed model analysis of covariance, with body length as a covariate, revealed large differences in eyespan among families at all loci (Table 6) and a significant effect of progeny genotype on eyespan phentoype for at least one sex at four loci. Progeny genotype explained 7% of the phenotypic variation in both male and female eyespan for CG33692, 9% of the variance in female eyespan for *corto*, 6.5% of the variance in male eyespan for 3531953:1, and 2% of the variance in female eyespan for *Eip75B*. A plot of mean eyespan by genotype reveals that longer glutamine repeats are associated with longer relative eyespan in both sexes for CG33692 (Fig. 2).

**Table 6:** Mixed model ANOVA on progeny eye span by progeny polyglutamine genotype and family

		Females				Males		
Source of variation	df	Var Comp%	F	P	df	Var Comp%	F	P
Band4.1 inhibitor LRP interactor (2)								
Family*	5	36.9	14.5	< 0.0001	5	53.6	31.7	< 0.0001
Genotype*	4	2.0	1.7	0.16	4	1.1	1.6	0.17
Error	177				202			
CG33692 (1)								
Family*	5	37.3	13.2	< 0.0001	5	38.6	18.2	< 0.0001
Genotype*	7	5.6	2.7	0.011	7	6.8	3.5	0.0013
Error	168				200			
Corto (2)								
Family*	5	33.7	10.5	< 0.0001	5	46.1	18.4	< 0.0001
Genotype*	9	4.9	2.1	0.035	9	1.1	1.3	0.23
Error	175				200			
Ecdysone-induced protein 75B (1)								
Family*	4	32.9	14.3	< 0.0001	4	43.3	19.2	< 0.0001
Genotype*	2	5.4	4.6	0.012	2	1.2	1.8	0.18
Error	134				119			
3531953:1 (X)								
Family*	4	47.7	16.2	< 0.0001	4	50.6	22.6	< 0.0001
Genotype*	5	-1.0	0.7	0.59	5	6.5	4.5	0.0049
Error	143				144			

*Family and genotype are random effects and body length is a significant (not shown) covariate in all models.

## Discussion

### Distribution, variation and evolution of amino acid repeat loci

By analyzing ESTs from genes expressed during head development we find that amino acid repeats typically consist of glutamine residues, are often found in transcription factors, and exhibit high levels of polymorphism. These results are consistent with previous studies [28,44,45], which have found that glutamine repeats were the most common codon repeat in *Drosophila*. Finding more glutamine than any other amino acid in repeats despite using a criterion that sampled far fewer repeats, i.e. less than 25% of repeats have 9 or more codons in *Drosophila *[46,47], suggests that glutamine may be particularly prone to repeat formation in flies. Indeed, Faux et al. [28] found that glutamine was also the most common amino acid among repeats in *Anopheles gambiae*, although it was only 1/6 as common as in *D. melanogaster*. In contrast to flies, glutamine repeats are only the seventh most common codon repeat in mammals and sixth most common in chickens [28].

Our discovery of extensive length polymorphism for glutamine repeats is consistent with reports suggesting that CAG and CAA trinucleotide repeats are particularly prone to replication error [19,48-50]. The similarity in measures of variation between coding and noncoding repeats suggests that similar mutational processes affect both types of repeats in stalk-eyed flies. Some of this variation may not have significant phenotypic consequences since genotypic frequencies inferred from PCR products amplified from a large sample of outbred flies matched Hardy-Weinberg expectations. However, five amino-acid repeat loci contained more homozygotes than expected (CG12104, CG31224, *Eip75B*, *M-spondin*, and *toutatis*) while two loci (*corto *and CG10082) exhibited more heterozygotes than expected. An overrepresentation of heterozygotes may indicate the presence of balancing selection [51]. An excess of homozygotes could be caused by null alleles, selection, inbreeding, or population substructure [51]. Because we found no evidence of excess homozygosity among microsatellite markers typed on the same individuals and flies were sampled from a population that has been maintained in the laboratory for over 10 years, inbreeding and population substructure are unlikely explanations for genotypic differences among loci. Selection, though, could explain deviations from Hardy Weinberg if either alleles or genotypes are under selection or near loci under selection. Genotypes from at least two of these loci (*corto *and *Eip75B*) explain variation in eyespan (see below). Because we estimated genotype frequencies using flies that had been selectively chosen to differ in relative eyespan, we would expect genotype frequencies to fail to conform to Hardy Weinberg expectations whenever loci are closely associated with eyespan.

Consistent with previous reports [10,17,45,52] we find that proteins with codon repeats tend to be transcription factors or associated with gene regulation more often than expected by chance. Although transcription factors exhibit sequence conservation in their DNA binding domains [53], codon repeats tend to occur in intrinsically disordered regions, which are poorly conserved [54,55]. Thus, length variation in codon repeats is less likely to influence transcription through DNA binding activity than through other mechanisms. Nevertheless, mutational studies on at least five different proteins with conserved codon repeats have demonstrated that alteration in amino acid repeat length alters protein function [29,56].

Replication slippage provides a plausible explanation for how variation in length is generated once an amino acid repeat has formed. The origin of a repeat is, however, less obvious and likely involves other genetic mechanisms, such as unequal crossing over or gene conversion. Recently, codon repeat abundance and length has been found to be greater in genes that exhibit alternative splicing, which are also enriched for poly-Q in *Drosophila *[29,56]. Alternative splicing may reduce constraints by exposing multiple forms of a protein to selection. Such relaxed selection may then permit invasion and expansion of a codon repeat. The relative lack of conservation among genes containing repeats of 9 residues or more in *T. dalmanni *and *D. melanogaster *(cf. Table 3) demonstrates that glutamine repeats can be gained or lost frequently over time, which for this species pair corresponds to about 70 MY [57]. This result contrasts with Mularoni et al. [29] who found 92 repeats 8 amino acids or longer in *Homo sapiens *that are conserved among Eutherians and reported that conserved codon repeats tend to be longer than noncoding repeats, indicative of positive selection. These results indicate that some of the evolutionary processes influencing the origin and evolution of amino acid repeats must differ between flies and vertebrates.

### Association between amino acid repeat loci and a sexually selected trait

To the extent that variation in codon repeat length alters regulation of transcription, SARPs provide a mechanism by which protein-coding regions may contribute to quantitative variation in phenotypic traits. In this study, we evaluate the possibility that length variation in loci with glutamine repeats influences the development of an unusual sexually selected and sexually dimorphic trait - relative eyespan in the stalk-eyed fly, *T. dalmanni*. Parental genotypes at five autosomal loci (*band4.1 inhibitor LRP interactor*, *ptip*, CG33692, *corto*, and *Eip75B *- Table 4) and one X-linked locus (3531953:1) predicted mean eyespan of progeny in at least one sex (Table 5). Several of these breeding value associations were confirmed by showing that amino acid repeat genotype predicted relative eyespan among siblings from families in which length variants were segregating at the candidate locus (Table 6). Progeny genotype explained significant variation in female eyespan for *corto *and *Eip75B*, in male eyespan for 3531953:1 and in both female and male eyespan for CG33692. Longer glutamine repeats in CG33692 are associated with longer relative eyespan in both sexes (cf. Fig. 2).

Four explanations are possible for these associations. First, associations between genotype and morphology might represent false positive results due to multiple testing or undetected population stratification. However, by first screening parent genotype against progeny breeding values and subsequently testing for associations between candidate loci genotype and progeny phenotype within nuclear families, we minimize spurious results. The apparent sex-limited genotype-phenotype associations at some loci, such as *corto *and *Eip75B*, suggest, though, that some associations are weak and deserve replication. Second, associations between genotype and morphology could result from epistatic interactions involving multiple loci. Additional breeding experiments need to be conducted to evaluate this possibility because only a limited number of genotypic combinations involving the candidate loci are represented in our breeding study. Third, associations between genotypes and morphology may be due to linkage disequilibrium. Studies on *Drosophila *have shown that linkage disequilibrium decays rapidly with respect to physical distance in large effective populations, but if populations are small, linkage disequilibrium may be present over longer distances [58]. It is possible, therefore, that some of the associations we found, such as that for CG33692, are caused by physical linkage to another gene that causes differences in eyestalk length. High-resolution mapping studies are needed to assess the plausibility of this suggestion. Finally, length variation in polyglutamine regions may directly influence head shape development. Ultimately, confirmation of any genotype-phenotype association will require some type of genetic manipulation, such as RNAi, that alters phenotypic expression during the appropriate developmental period.

If any of these associations do reflect causal relationships, we would expect the known biological functions of the candidate genes to be consistent with modification to head and eye development. Accordingly, three of the five candidate genes have known phenotypic effects on eye development. Unfortunately, neither the molecular function nor the biological process for CG33692 is known [59]. Two of the other candidate genes, *corto *and *Eip75B*, are known to exhibit transcription factor activity. Specifically, *corto *exhibits RNA polymerase II transcription factor activity, and protein binding [60,61]. Furthermore, *corto *acts alternatively as an *enhancer of trithorax *and an enhancer of *polycomb*. These two groups of proteins are responsible for maintaining *homeotic *(*Hox*) gene expression throughout development [62] and *homeobox *genes are required for *Drosophila *visual system development [63,64].

*Ecdysone-induced protein 75B *exhibits transcription factor activity as well as regulation of transcription [65] and oogenesis [66] and is part of a small group of genes whose early expression is essential for *ecdysone *induced changes during developmental transitions [67]. Ecdysteroids trigger major developmental transitions such as larval molting and metamorphosis in flies [68]. Furthermore, *Eip75B *affects eye formation in flies and moths [69,70].

The EST 3531953:1 has not yet been identified but the amino acid sequence shares structural characteristics with *tousled-like kinases *(*Tlk*s). *Tlk*s are a family of serine/threonine kinases that are involved in the cell cycle [71-75], chromatin assembly [72,75], DNA repair [76], transcription [77], and chromosome segregation [78,79]. *Tlk *has been directly implicated in spermatogenesis [74] and expression studies have documented a loss of *tlk *expression results in cell cycle arrest and apoptosis [72]. In *D. melanogaster*, overexpression of *tlk *also results in change in the texture of ommatidia and a decrease in eye size [72].

Because 3531953:1 is located on the X chromosome in *T. dalmanni *and exhibits structural similarity to *tlks*, which influence spermatogenesis and eye development, this gene is also a potential candidate for sex chromosome meiotic drive in stalk-eyed flies [40,41]. Sex chromosome meiotic drive typically occurs by differential survival of sperm [80,81] reviewed in: [82]. Male stalk-eyed flies that produce broods composed predominantly of daughters also show evidence of abnormal sperm development consistent with degenerate Y-bearing sperm [42,83]. Furthermore, the X chromosome explains over 30% of the variation in relative eyespan between lines selected for increased or decreased eyespan [43] and males that lack drive X chromosomes have longer eyestalks [42]. Thus, the possibility that 3531953:1 may provide a mechanistic link between meiotic drive and eyestalk length merits future study.

## Conclusions

The effect that coding or regulatory sequence evolution has on the evolution of morphology is still contentious. Single amino acid repeat polymorphisms (SARPs) have been proposed as a genetic mechanism that can generate morphological variation [10,17,30,84-88]. This study provides several lines of support for these claims. SARPs are over-represented among genes that contain repeated glutamine residues and influence regulation of transcription. We find that glutamine repeats exhibit levels of variation comparable to anonymous dinucleotide microsatellites and can be independently gained or lost between fly species. Genotypes at five loci independently explain variation in the phenotype of a sexually selected trait, eyestalk length, and current annotation for four of those genes is consistent with a biologically important function in eyestalk development. While these putative associations are intriguing, confirmation must await fine scale mapping studies and genetic manipulations to demonstrate that allelic variation alters phenotypic expression.

## Methods

### Identification of repeat genes

Because EST libraries are efficient for amino acid repeat discovery [89], we searched amino acid sequences from a *T. dalmanni *EST database [35] for strings of nine or more consecutive amino acids. This search identified 252 ESTs. Of these, 88 carried terminal lysine or phenylalanine repeats and were excluded as cases of poly-A tails. Of the remaining 164 ESTs, 120 were identifiable on the basis of BlastX (< 1e^-9^) similarity to a protein in *D. melangaster *and represented 98 unique genes [35]. Because they are based on partial gene sequence, some annotations may change with additional sequence data.

To compare the distribution of single amino acid repeats between *T. dalmanni*, and *Drosophila melanogaster*, we created a protein database for *D. melanogaster *that contained only homologous regions to the *T. dalmanni *EST database as determined by a BlastX alignment output. A search of this database revealed 343 loci with at least one repeat containing 9 or more amino acids. Then, to determine if repeats form at random with respect to amino acid we compared the frequency of amino acids in the *T. dalmanni *EST database or the homologous regions for *D. melanogaster *to the frequency of each repeat using a chi-squared goodness of fit test. For the *T. dalmanni *repeat genes, we then used GeneMerge with Bonferroni adjustment [39] to determine if the molecular function represented a nonrandom sample of the putative protein-coding genes in the EST database.

### Repeat length variation

To assess variation in repeat length in *T. dalmanni*, we attempted to genotype at least 51 male and 51 female flies reared from a large, outbred population of *T. dalmanni *originally collected in 1999 near Ulu Gombak in peninsular Malaysia [40,41] and subsequently maintained as a single population of over 200 individuals with approximately three overlapping generations per year. These flies were used as parents in the association study described below. We used Primer3 [90] to design polymerase chain reaction (PCR) primers that would amplify the repeat and flanking regions for 42 of the original 64 loci containing glutamine repeats. These 42 were chosen because they contained sufficient high complexity flanking regions around repeat regions to design primers. Nine of these primer sets either did not amplify a fragment of the anticipated size or produced inconsistent banding patterns. In addition, the primers for one locus (*hairy*) produced a fragment that was too large to genotype easily. The remaining primer sets produced reliable PCR products for 32 loci and form the basis of this study.

PCR was carried out in 10 μl reactions containing 40 ng template DNA, 1× reaction buffer, 2.5 mM MgCl_2_, 0.20 mM dNTPs, 2.5 pmol of each primer, and 0.5 U *Taq *DNA polymerase. Each amplification reaction was initiated at 95°C for 5 min; followed by 35 cycles of 95°C for 45 s, annealing temperature for 1 minute, and 72°C for 45 s; and terminated at 72°C for 10 min. Primer sequences and annealing temperatures for each locus are listed in Additional File 1, Table S1. PCR products were labeled either with a fluorescent M13 primer according to the protocol outlined in Schuelke [91] or created with primers containing fluorescently labeled nucleotides. Labeled PCR products were genotyped on an ABI 3730 DNA analyzer and products were sized using ROX500 and scored with GeneMapper 4.0 according to manufacturer (Applied Biosystems) protocols.

In addition to scoring loci for amino-acid repeat length, we also genotyped eight autosomal (chromosome 1: ms262Z, ms336, ms392, ms398; chromosome 2: ms90, ms249, ms301, ms422) and eight X-linked (ms70, ms71, ms106, ms125, ms167, ms244, ms395, ms478) noncoding dinucleotide microsatellites [38], whose genomic location had been previously determined by linkage mapping [42]. We determined chromosomal association for each codon repeat locus on the basis of significant chi-squared contingency tests between it and one or more microsatellite markers. In addition, we calculated and compared several measures of genetic variation (see below) to assess the possibility that a common mutation process, such as replication slippage, could account for length variation in both types of repeats.

### Sequence analysis

To verify that variation in PCR product length was due to differences in the number of amino acids in a repeat we used Sequencher v. 4 to align and compare all loci for which we had three or more EST sequences. In addition, to confirm that amplification products contained amino acid repeats, at least one PCR product was sequenced for each locus. Sequences were obtained using the ABI Prism Big Dye Terminator Cycle Sequencing Ready Reaction kit using an ABI 3730 automatic DNA sequencer according to the manufacturer's specifications (Applied Biosystems). Sequences were cleaned, edited, and aligned using Sequencher v. 4.

### Comparison of glutamine repeats between species

To determine if glutamine repeats are present in similar locations in both *T. dalmanni *and *D. melanogaster*, and therefore likely shared by a common ancestor, we used a protein database for *D. melanogaster *that contained only homologous regions to the *T. dalmanni *EST database as described above. We then searched both databases for all occurrences of more than eight consecutive glutamine residues and counted, in the other species, the total number of glutamines in the region homologous to the polyglutamine repeat. Because only a subset of the *T. dalmanni *EST consensus sequences have homologous sequence in *D. melanogaster *that is incorporated into the Blast alignment, this search resulted in fewer total polyglutamine repeats than were identified in the search of the entire EST database. However, it is important to note that analysis of these homologous gene region databases in both species eliminates much of the ascertainment bias that can potentially confound comparison between the species.

### Estimating association with eyespan

To determine if variation in any of the SARP loci is associated with eyespan variation, we conducted a two-generation association experiment using an outbred laboratory population of *T. dalmanni*. This population was originally collected near the village of Gombak in peninsular Malaysia in 1999 and subsequently has been maintained with a population of over 200 individuals. In the first generation, we mated flies assortatively on the basis of relative eyespan in order to include alleles with extreme effects. We measured 314 females and 273 males and then selected 46 pairs with the largest and 46 pairs with the smallest eyespan to body length ratio for breeding. We used CO_2 _anesthesia to capture an 11× video image of each fly resting on its thoracic and orbital spines. Eyespan from the outer edges of the ommatidia, body length from the face to the wing tip, and thorax width were then measured at a resolution of 50 pixels/micrometer using Scion Image v1.59.

Breeding pairs were kept in 2.5 l clear plastic jars and 50 ml of pureed corn was provided as food and oviposition substrate twice each week for three weeks to allow progeny to develop under low competitive conditions. Fifty-one of the 92 pairs produced 20 or more progeny. After eclosion progeny were frozen at -20°C. Eyespan, body length and thorax width were measured from 10 male and 10 female progeny and used to calculate breeding values in eyespan, after adjusting for body length, for each pair. A random effects analysis of variance was used to confirm that heritable variation in eyespan was present in this sample (results not shown).

To identify loci with potential effects on relative eyespan we tested for an association between parental genotype and offspring breeding value. DNA was extracted from each parent using Chelex [92]. Parental genotypes were obtained for 47 of the 51 families at amino acid repeat loci and nocoding microsatellites [38]. For each locus we calculated observed heterozygosity, allelic diversity (i.e. expected heterozygosity) and tested for deviations from Hardy-Weinberg equilibrium expectations using a chi-squared goodness of fit test.

For autosomal loci we used analyses of covariance on eyespan, with body length as a covariate, to determine if son or daughter breeding values differed among parental genotypes at each locus. We conducted similar analyses for X-linked loci except that male and female parents were analyzed separately to account for the different patterns of inheritance of X-linked loci. Loci with significant (P ≤ 0.01) effects of parental genotype for either sex were selected for additional analysis to determine if progeny phenotypes differed among progeny genotypes within families. For each significant locus we examined the parental genotypes and attempted to identify at least five families in which the parental alleles would segregate such that the progeny would carry genotypes that would be expected to differ in eyespan. Eyespan, body length, and thorax width were then measured on a total of 956 offspring from 19 families. Progeny DNA was isolated using Chelex [92] and amplification was carried out as described above. A mixed model analysis of covariance was performed on progeny eyespan in which family and genotype were random effects and body length was a covariate for flies of each sex. We estimated variance components using restricted maximum likelihood to indicate how much of the variation in relative eyespan could be attributed to genetic variation within families.

We used JMP v5.0.1.2 (SAS Institute, 2003) for all statistical analyses.

## Authors' contributions

LB conducted all genotyping and sequencing and drafted the manuscript, MP did the breeding and phenotypic measuring for the association study, RB helped to conceive of the study, identify homologous gene regions, and edit the manuscript, and GW guided the study from origin to completion, conducted many of the statistical analyses, and edited the manuscript. All authors have read and approved the final manuscript.

## Supplementary Material

Additional file 1 **Primer sequences and annealing temperatures for each locus**. This table contains the forward and reverse primers and annealing temperatures that were used in polymerase chain reactions. Product size range is also provided. Click here for file
