# Supplementary material for: Length polymorphism and head shape association among genes with polyglutamine repeats in the stalk-eyed fly, Teleopsis dalmanni
Source: BMC Evol Biol. 2010 Jul 27;10:227. doi: 10.1186/1471-2148-10-227 (PMC3055267; doi:10.1186/1471-2148-10-227)
Supplement: Additional file 1 — Primer sequences and annealing temperatures for each locus. This table contains the forward and reverse primers and annealing temperatures that were used in polymerase chain reactions. Product size range is also provided. [file 1471-2148-10-227-S1.DOC]

Additional File 1, Table S1. Primer sequences and annealing temperatures for each locus.

| Locus | Primer Sets (5’-3’) | T_a_ (˚C)* | Range size (bp) |
| --- | --- | --- | --- |
| Band4.1 inhibitor LRP interactor | F: GGT GGA AGT GCG GG  R: G GTG GGA CTG TGA ATG GTG ATG ATG TGC | 68.5* | 301-319 |
| Bifocal | F: CAT TAC ACG CCG C  R: G GAG ACA ATT GTG GTT CAG | 62.9* | 119-134 |
| Bunched | F: FAM/AGC GGT CGC AGG AGG CAT G  R: CGC ACC ACA ATC GAC AGT TTA CAT CAT TGG TCC | 68 | 185-191 |
| Cap-n-collar | F: GAT GCT GTA AAT GGT ATT GCT G  R: C CAA TTC GTA CAG TGT AGC AG | 68.5* | 359-362 |
| CG10082 | F: GTT ACT GGT GAT AAA TCA C  R: G GAC TCA AAT GCA CTA GC | 62.9* | 196-212 |
| CG10321 | F: GTA CGT GTG GTA ATA GAG GAG CC  R: G TCG AGA CGT GTT ACC GCT AAC | 68* | 529-538 |
| CG10435 | F: GCA ATA CAC AAT GAT GTT GAA C  R: G CCG ACA AGA TGA GTA ATA TGT ATA GGA C | 64.3* | 205-253 |
| CG12104 | F: CTG ACA CTT TAC GTG CCG  R: G GTA TTG TGA ATA CAG CCG G | 68* | 189-192 |
| CG17265 | F: GGA GGA AGA ACG TTT AAC  R: C CGA TAC GAT CTC GTT CCA G | 64.3* | 263 |
| CG31064 | F: GCA ATG GTG CAC  R: G GCA TGT GCT GC | 60.8* | 164-182 |
| CG31224 | F: TGA ATT CGC ATG TAC C  R: C AGC TGC AAT GGT GGT GGA G | 68* | 494-512 |
| CG33692 | F: CCA TTT GAT TGG CTT GGC  R: G CAG CAG AAG ATG AAG ATG | 67.5* | 310-319 |
| CG34347 | F: GGA ATT TAT GCG TCC CG  R: C GGA TCA GCA GTT TGT GCG | 68* | 156-183 |
| CG42389 | F: CAT CAT GCA CAC CAT GG  R: G CCA GCA CTA CTG CCA ATG | 67* | 260-272 |
| CG8668 | F: CAC TGT CAT CAT CAG TAG  R: G GCA TAA AAG TTT GCT TCG | 64.3* | 173-188 |
| CG4409 | F: GAA TCC GTG ATG GAT AC  R: G ATT CCT CTT CGT CGG ATG | 64.3* | 205-214 |
| Corto | F: CCA CAA ATT CTG CTG CAA C  R: G AGG CGA CTG TGG TAT GAC | 68* | 493-514 |
| Cryptocephal | F: FAM/CCAGTTCAAATTGTAACCAACG  R: TCGACAATTTGCATTTCACGTGC | 50 | 193-227 |
| Cyclin-dependent kinase 8 | F: GGT TGT CCT ATA CCG TAC  R: C GTA CAC GTT TCG CTT C | 60.8* | 225 |
| Dachshund | F: GTC CAC CAC ACA GTA AAC AC  R: C GTC CAT TGG TAC GTT CAT CG | 62.9* | 227 |
| Dorsal switch protein 1 | F: CCG AAT ATA AGA CCA GCG  R: C ATC ATC ATG TTC TTC ATG ATG C | 66* | 181-222 |
| E5 | F: GAA ATA GAT GCG GAA GAA C  R: C AGA ATG CAA ACG CCA ATG | 68* | 406-409 |
| Ecdysone-induced protein 75B | F: GCA ATG AGC GGT GTT AGG AC  R: G AGC AAC CAA CAA CGA TGT G | 67.5* | 407-410 |
| M-spondin | F: GGA CAG TAT TGC AGG AG  R: G CGG GTA CAT TAA ATT CAT CG | 64.3* | 235-283 |
| Mastermind | F: GGA TGC AGG AGG TCT TC  R: G ATG CCG TTG ATG TTG TTC C | 68* | 520-526 |
| Mediator complex subunit 26 | F: CTA ATT GTG ACC AAC TAG CAC  R: G GTC CAA TTC CAT CTG GAA C | 68* | 318 |
| Ptip | F: GAG TTC TAC ATT GGC CAG TC  R: G CGG CCG TTG CTG TAA TG | 68* | 199-214 |
| Sine oculis-binding protein | F: CGA AAT TCA AGA TTA CAC GAT TAC  R: G GAA ATC ACA ATC TAT AAT GCG | 66* | 229 |
| SRPK | F: CAC TAT GAA TGT CGA TTG C  R: C ATA GGA AGA ACT TCG TTC | 61.7* | 154-172 |
| Tenascin major | F: AGTA AAA CGA CGG CCA GT C CTG GAC CCG GTT C  R: C GTA GTC GTA ATG TGA CGG | 68.5* | 200-206 |
| Toutatis | F: CTA CAA TAC TGT AAC ACC TC  R: G TAG TAA TGA TGG TGA TG | 64.3* | 154-166 |
| 3531953:1 | F: CAT TTG CTA AAT TAT CGC  R: G CAC TTA ATT TGG ACA GTG ATG | 64.3* | 288-318 |

*annealing temperatures include M13 tags; F M13: AGT AAA ACG ACG GCC AGT; R M13: CAG GAA ACA GCT ATG AC
